# Inhibition of APN/CD13 leads to suppressed progressive potential in ovarian carcinoma cells

**DOI:** 10.1186/1471-2407-7-140

**Published:** 2007-07-27

**Authors:** Mikio Terauchi, Hiroaki Kajiyama, Kiyosumi Shibata, Kazuhiko Ino, Akihiro Nawa, Shigehiko Mizutani, Fumitaka Kikkawa

**Affiliations:** 1 Department of Obstetrics and Gynecology, Nagoya University, Graduate School of Medicine, Tsuruma-cho 65, Showa-ku, Nagoya 466-8550, Japan

## Abstract

**Background:**

Aminopeptidase N (APN/CD13), a 150-kDa metalloprotease, is a multifunctional cell surface aminopeptidase with ubiquitous expression. Recent studies have suggested that APN/CD13 plays an important role in tumor progression of several human malignancies. In the current study, we investigated the role of APN/CD13 in ovarian carcinoma (OVCA) progression.

**Methods:**

We first examined the expression of APN/CD13 at the protein level in a variety of OVCA cell lines and tissues. We subsequently investigated whether there was a correlation between APN/CD13 expression and invasive potential of various OVCA cell lines. Moreover, we investigated the function of APN/CD13 in OVCA cells using bestatin, an APN/CD13 inhibitor, or transfection of siRNA for APN/CD13.

**Results:**

We confirmed that APN/CD13 was expressed in OVCA tissues and cell lines to various extents. There was a positive correlation between APN/CD13 expression and migratory potential in various OVCA cell lines with accordingly enhanced secretion of endogenous MMP-2. Subsequently, we found a significant decrease in the proliferative and migratory abilities of OVCA cells after the addition of bestatin or the inhibition of APN/CD13 expression by siRNA. Furthermore, in an animal model, daily intraperitoneal administration of bestatin after inoculation of OVCA cells resulted in a decrease of peritoneal dissemination and in prolonged survival of nude mice.

**Conclusion:**

The current data indicate the possible involvement of APN/CD13 in the development of OVCA, and suggest that clinical use of bestatin may contribute to better prognosis for ovarian carcinoma patients.

## Background

Aminopeptidase N (APN/EC 3.4.11.2) is a type II membrane-bound metalloproteinase expressed on various cell types, such as kidney, intestinal epithelium, liver, placenta, and lung cells [1-3]. APN is also a cell surface aminopeptidase that was originally characterized as a myeloid marker[4]. APN/CD13 activates or inactivates bioactive peptides on the cell surface by cleaving them enzymatically and regulates their availability to adjacent cells. Importantly, recent reports have indicated that APN/CD13 has a variety of functions, including roles in inflammatory and immunological responses, signal transduction, antigen processing, neuropeptide and cytokine degradation, and extracellular matrix degradation [5-9]. In addition, a number of studies have provided evidence that APN/CD13 may play a role in tumor progression by regulating processes such as cell-cell contact, proliferation, tumor invasion, and angiogenesis [5,10-14]. Furthermore, a recent study showed that APN/CD13 was involved in the protection of leukemic cells against apoptosis[15].

Epithelial ovarian carcinoma (OVCA) is a major cause of death among gynecological malignancies [16]. Since OVCA frequently remains clinically silent, the majority of patients with this disease have advanced intraperitoneal metastatic disease at diagnosis [17]. The biological behavior of this carcinoma is associated with clinicopathological parameters, including International Federation of Gynecologists and Obstetricians (FIGO) stage, tumor grade, and histological type. Treatment for advanced OVCA is difficult because of both the inability to completely resect diffuse tumors on the peritoneal surface and the eventual resistance of the tumor cells to chemotherapy.

We have investigated the molecular mechanism of OVCA progression. Especially, our recent reports focused on the involvement of cell surface aminopeptidases such as dipeptidyl peptidase IV (DPPIV/CD26) and neutral endopeptidase 24.11 (NEP/CD10) in the peritoneal progression of this carcinoma, and demonstrated that overexpression of DPPIV or NEP in highly invasive OVCA cells significantly decreased peritoneal dissemination and increased survival time in a mouse model [18,19].

In the current study, we investigated the possible role of APN/CD13 in OVCA progression. We first examined the expression level of APN/CD13 in various OVCA cell lines. Subsequently, to clarify the cellular roles of APN/CD13 in OVCA, we investigated the progression of OVCA *in vitro *and *in vivo *using bestatin, an APN/CD13 inhibitor, or siRNA specific for APN/CD13. The possible function of this enzyme as an inducer of OVCA progression is proposed.

## Methods

### Cell culture

Seven human OVCA cell lines (SKOV-3, HRA, ES-2, HEY, NOS2, NOS4, and TAOV) were cultured and maintained as described previously [19]. ES-2 and HEY cells were purchased from the American Type Culture Collection (ATCC) and were maintained in RPMI-1640 (Sigma) supplemented with 10% fetal calf serum (FCS) and penicillin-streptomycin. These cells were incubated at 37°C in a humidified atmosphere containing 5% CO_2_.

### Enzyme activity assay

APN/CD13 enzyme activity was measured spectrophotometrically using L-leucine-p-nitroanilide (Peptide Institute, Inc.) as an APN/CD13 substrate. Whole-cell suspensions were prepared in test tubes, and then washed with phosphate-buffered saline (PBS). Thereafter, 5 × 10^5 ^cells were resuspended in 200 μl of PBS in each well of a 96-well microtiter plate, and the substrate was added (final 1.6 mM). APN/CD13 enzyme activity was estimated by measuring the absorbance at 405 nm using a microplate reader (Labsystems, Multiskan Bichromatic) every 15 min during incubation at 37°C.

### Flow cytometric analysis

Fluorescence-activated cell sorting (FACS) was performed to quantify the expression level of APN/CD13 on the cell surface of OVCA cells. Then, the cells were incubated with phycoerythrin-conjugated monoclonal antibody specific for APN/CD13 (BD Pharmingen, CD13mAb clone: WM15, San Diego, CA) for 30 min at 4°C, and washed three times with PBS. FACS data were acquired on a FACS Calibur (Becton Dickinson, San Jose, CA), and analyzed using CELL Quest software (Becton Dickinson).

### Immunohistochemical staining

Fourteen tissue samples of OVCA were obtained with informed consent from patients who were surgically treated at Nagoya University Hospital. All samples were fixed in 10 percent formalin and embedded in paraffin, and sections were cut at a thickness of 4 μm. For heat-induced epitope retrieval, deparaffinized sections in 0.01 M citrate buffer were treated three times for 5 min at 90°C at 750 W using a microwave oven. Immunohistochemical staining was performed using the avidin-biotin immunoperoxidase technique (Histofine SAB-PO kit, Nichirei, Tokyo, Japan). Endogenous peroxidase activity was blocked by incubation with 3% H_2_O_2_, and non-specific immunoglobulin binding was blocked by incubation with 10% normal rabbit serum. As a first antibody for APN/CD13 staining, anti-APN/CD13 mAb (Novocastra, CD13mAb clone: 38C12, Newcastle, United Kingdom) was used at a dilution of 1:100.

### Inhibition of APN/CD13 by small interfering RNA

We designed and purchased two different siRNA duplexes of APN/CD13, si-CD13 (sense, 5'-CACCUUGGACCAAAGUAAA-3'), and si-CD13-2 (sense, 5'-GAAAUGCCACACUGGUCAA-3') from Qiagen (Tokyo, Japan). Nonspecific control siRNA duplexes (si-cont) (sense, 5'-CUGGAUUGUAGGAAGUACCTT-3') with the same GC content as APN/CD13 siRNA were purchased from Takara (Tokyo, Japan). The siRNA was transfected into ES-2 cells at a final concentration of 80 nmon/L using a GenePorter-2 (Genlantis, San Diego, CA) according to the manufacturer's protocol.

### Cell doubling time

Cells growing in log phase were harvested and plated in a 6-well plate at 7.5 × 10^4 ^cells/well in RPMI1640 +10% FCS. The number of cells was counted at 24, 48, 72, and 96 h. The cell doubling time in log phase was determined in three separate experiments.

### Cytotoxicity assay: trypan blue dye-exclusion test

The trypan blue dye-exclusion test was used to determine drug-mediated cytotoxicity as described previously[20]. The following preparations were assessed for their ability to inhibit cell growth. Briefly, target tumor cells were resuspended in medium at 25 × 10^4 ^cells/mL after verifying cell viability by the trypan blue dye exclusion test (Sigma Chemical Co.). First, cells were incubated for 24 h at 25 × 10^4 ^cells/well using 6-well, flat-bottomed plates. Then, various concentrations of drug samples were added for a further 24 h. Each plate was incubated for 72 h at 37°C in a 5% CO_2 _atmosphere. Following the incubations, 100 μL of the trypan blue dye was added to 100 μL of cell suspension, and viable and dead cells were counted. To estimate the cytotoxicity, control cell groups without any drug treatment were compared with drug-treated cell groups, and the experiments were repeated three times.

### Cell proliferation assay

The cell proliferation assay was performed as described previously [18]. Cells were seeded in triplicate in 96-well plates at a density of 2000 cells in a volume of 200 μl of RPMI1640 containing 10% FCS, and cultured for 1 to 4 days in the presence or absence of bestatin (Nihon-Kayaku, Tokyo, Japan). Cell viability was assayed by a modified tetrazolium salt 3-(4,5-dimethylthiazol-2-yl)-2,5-diphenyltetrazolium bromide assay using a Cell Titer 96 Aqueous One Solution Cell Proliferation Assay kit (Promega Corp., Tokyo, Japan) according to the manufacturer's instructions. Absorbance was measured at 492 nm using a microplate reader (Multiskan Bichromatic; Labsystems Helsinki, Finland). Furthermore, we examined the effect of siRNA transfection on the proliferation of ES-2 cells. ES-2 cells were seeded in 96-well plates at 1500 cells/well in 200 μl of culture medium containing 10% FCS. After 24 hours, the medium was replaced by fresh RPMI 1640 containing 5% FCS and transfection of the siRNA duplexes (si-cont and si-CD13) was performed in same the 96-well plate using a GenePorter-2 (Genlantis, San Diego, CA). Cell proliferation was then evaluated using a 5-day modified MTT assay as described above.

### In vitro migration assay

Cell migration was assayed in 24-well Transwell cell culture chambers (Costar). Cells were suspended in the upper chamber at a final concentration of 50 × 10^4^/ml in 200 μl of RPMI 1640 in the presence or absence of bestatin (0, 10, 100, or 200 μg/ml). The lower chamber contained 700 μl of RPMI 1640 supplemented with 10% FCS. After 8 hour of incubation, the tumor cells remaining on the upper surface of the filters were removed by wiping with cotton swabs, and the migrated cells on the lower surface were stained with May-Grünwald Giemsa staining. The number of cells on the lower surface of the filters was counted under a microscope at a magnification of 200. In addition, we examined the effect of siRNA transfection on the migration of ES-2 cells. ES-2 cells were seeded in 10-cm dishes in RPMI1640 containing 10% FCS. After reaching 50% confluency, the medium was replaced by fresh RPMI 1640 containing 10% FCS, and transfection of siRNA (si-cont or si-CD13) was performed using a GenePorter-2 (San Diego, CA). Sixty hours after transfection, the cells were trypsinized and pelleted. Subsequently, the cells were re-plated in the upper chambers of Transwell plates at a density of 50 × 10^4^/ml in 200 μl of RPMI 1640. The lower chamber contained 700 μl of RPMI 1640 supplemented with 10% FCS. The subsequent procedures were the same as described above. We performed four individual experiments in which this assay was performed in triplicate.

### MMP-2 and VEGF quantification by ELISA

OVCA cells were seeded into 6-well culture dishes, and incubated in culture medium. After achieving subconfluency, cells were washed with RPMI1640 containing 1% FCS, and incubated for 48 hours. After incubation, the culture supernatants were tested using MMP-2 ELISA kits (R&D Systems, Minneapolis, MN) according to the manufacturer's protocol. Experiments were performed in triplicate. In addition, we examined the effect of siRNA transfection on MMP-2 and VEGF expression in ES-2 cells. ES-2 cells were seeded in 6-well dishes in RPMI1640 containing 10% FCS. After the cells reached 50% confluency, the medium was replaced by fresh RPMI 1640 containing 10% FCS, and transfection of siRNA (si-cont or si-CD13) was performed using a GenePorter-2 (Genlatis, San Diego, CA). Twenty-four hours after transfection, the medium was replaced by fresh RPMI1640 containing 1% FCS. After 48 hours of incubation, the culture supernatants were collected. The subsequent procedures were the same as described above using MMP-2 and VEGF ELISA kits (R&D Systems, Minneapolis, MN).

### *In vivo *studies

Five-week-old female nude mice (BALB/c) were provided by Japan SLC (Nagoya, Japan). The treatment protocol followed the guidelines for animal experimentation adopted by Nagoya University. HRA or SKOV-3 cells (5 × 10^6 ^cells/0.5 ml of medium/mouse) were injected i.p. to examine their peritoneal metastatic potential. The survival time was examined with or without treatment with bestatin.

Intraperitoneal (i.p.) administration of bestatin (20 mg/kg body weight) was initiated 24 hours after tumor inoculation, and was continued daily for 28 days (n = 7). In the control group (n = 7), vehicle only was administered in the same manner. Survival time was compared between these two groups. To confirm same results, this experiment was repeated twice.

### Statistical analysis

For data of *in vitro *and *vivo *experiments, statistical comparisons among groups were performed by the Student's *t*-test or ANOVA with Bonferroni corrections. Data are expressed as mean ± SD. P < 0.05 was considered significant.

## Results

### APN/CD13 expression in OVCA tissues and cell lines

**Figure 1:**
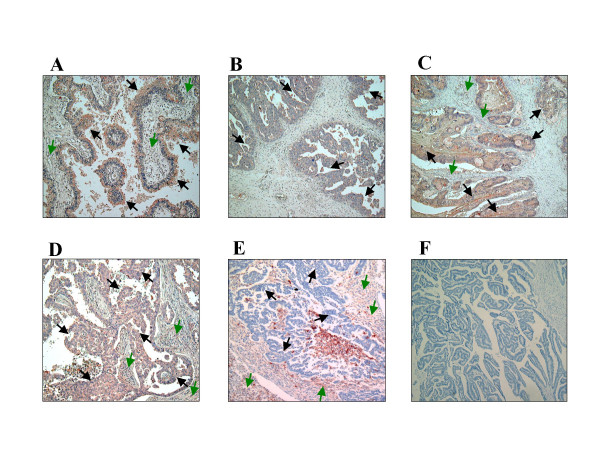
APN/CD13 expression in OVCA tissues A, B, C, D and E; Immunohistological staining of APN/CD13 in surgically resected OVCA tissues of 14 patients representing four different histological types using APN/CD13 specific Ab. A-D; APN/CD13 was expressed intensely in tumor cells but only slightly in stromal cells. A; clear cell carcinoma (case 7), B; endometrioid adenocarcinoma (case 8), C; mucinous cystadenocarcinoma (case 5), D; serous cystadenocarcinoma (case 12), E; serous cystadenocarcinoma (case 2), APN/CD13 was intensely expressed in stromal cells but was almost undetectable in tumor cells. F; APN/CD13-negative control of serous cystadenocarcinoma. Black arrows indicate tumor cells expressing APN/CD13; green arrows indicate stromal cells expressing APN/CD13.

**Figure 2:**
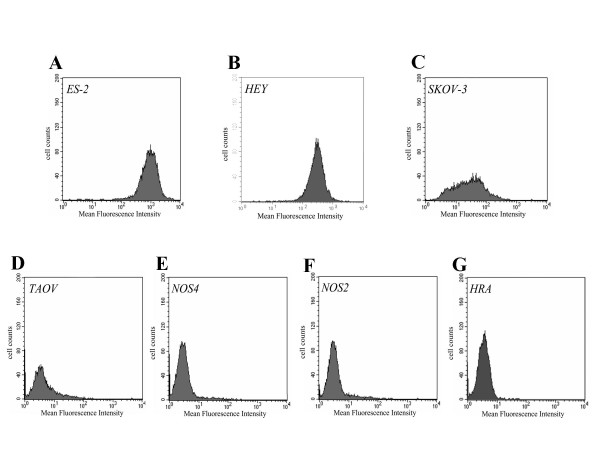
APN/CD13 expression in various OVCA cell lines, as estimated by FACS analysis *A*; ES-2 cells, *B*; HEY cells, *C*; SKOV-3 cells, *D*; TAOV cells, *E*; NOS-4 cells, *F*; NOS-2 cells and *G*; HRA cells.

We first examined APN/CD13 expression in 14 surgically resected OVCA samples. APN/CD13 was predominantly expressed in tumor cells of OVCA tissues, although the intensity of immunohistochemical staining varied from tumor to tumor. (Fig. 1A-D). However, in some tumors, APN/CD13 was also expressed in stromal cells (Fig. 1E). The clinicopathological data of the OVCA tissues examined are summarized in Table 1. In addition, we found a small number of APN/CD13-positive blood vessels in all OVCA tissues although the vascular density did not seem to be associated with the APN/CD13 expression in the tumor cells. Fig. 2 shows the varying levels of APN/CD13 expression in OVCA cell lines, as measured by FACS analysis. SKOV-3, ES-2 and HEY cells were intensely positive for APN/CD13, while in other cell lines, the level of APN/CD13 expression was lower, which was consistent with data obtained by enzyme activity analysis (data not shown).

**Table 1:** Summary of patient characteristics and APN/CD13 expression

**Case**	**PT**	**Histological type**	**Tumor grade**	**APN/CD13 (tumor expresion)**
1	1c	endometrioid	1	N
2	3c	serous	2	N
3	3c	mucinous	2	P
4	3c	serous	3	N
5	1a	mucinous	1	P
6	3c	mucinous	1	N
7	1c	clear cell	*N.D.*	P
8	2c	endometrioid	2	P
9	1c	clear cell	*N.D.*	P
10	2c	serous	3	P
11	1c	clear cell	*N.D.*	N
12	3c	serous	2	P
13	1c	endometrioid	1	N
14	1a	mucinous	1	N

N.D.: Not determined, N: Negative, P: Positive

### Correlation among APN/CD13 expression, migratory potential, MMP-2 expression and cell doubling time

Table 2 shows the correlation among mean fluorescence intensity of APN/CD13 in FACS, migratory potential, MMP-2 expression, and cell doubling time in these OVCA cell lines. The APN/CD13 expression in these cell lines was positively correlated with their migratory potential and with MMP-2 expression. In contrast, there was no obvious correlation between the expressions of APN/CD13 and doubling time among these OVCA cell lines. In addition, the morphology of APN/CD13-expressing cell lines tended to show a long spindle/bipolar pattern resembling fibroblasts, in contrast to that of non-APN/CD13-expressing cell lines, which show an epithelioid pattern.

**Table 2:** Correlation among APN/CD13 expression, migratory potential, and MMP-2 activity in OVCA cell lines

	**APN/CD13^*a *^expression**	**Migratory^*b *^cell counts**	**MMP-2^*c *^activity**	**Doubling time (h)**	**Morphology**
ES-2	987.3	168 ± 22	34.2 ± 4.8	18.6 ± 3.7	Fibroblastic
HEY	692.6	153 ± 19	31.5 ± 5.1	22.3 ± 2.4	Fibroblastic
SKOV-3	65.2	125 ± 23	23.9 ± 3.9	18.6 ± 4.7	Fibroblastic
HRA	4.5	129 ± 7	1.9 ± 0.9	17.4 ± 2.6	Fibroblastic
TAOV	7.4	22 ± 8	3.2 ± 2.1	20.2 ± 3.2	Epithelioid
NOS2	5.0	11 ± 4	1.6 ± 1.2	18.9 ± 3.7	Epithelioid
NOS4	3.8	17 ± 6	1.7 ± 0.9	19.4 ± 3.1	Epithelioid

*a *Mean fluorescence intensity by FACS analysis

*b *By Transwell migration assay × 200 HPF

*c *By ELISA assay in conditioned medium (ng/ml)

### The effect of bestatin on proliferation and migration of APN/CD13-expressing OVCA cells in vitro

**Figure 3:**
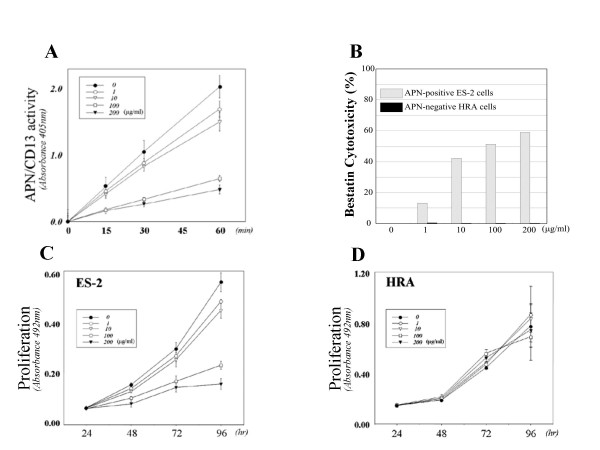
Differential effects of bestatin on cell proliferation of ES-2 and HRA cells. A; Decrease of APN/CD13 enzyme activity by bestatin in a concentration-dependent manner in ES-2 cells, which expressed APN/CD13. More than 100 μg/ml of bestatin significantly inhibited APN/CD13 enzyme activity in ES-2 cells (p < 0.01). B; Cytotoxicity of bestatin was evaluated by trypan blue dye-exclusion test for APN/CD13-positive ES-2 cells and APN/CD13-negative HRA cells. C; Bestatin dose-dependently suppressed cell proliferation of ES-2 cells, which strongly expressed APN/CD13. More than 100 μg/ml of bestatin significantly inhibited the proliferation of ES-2 cells (p < 0.01). D; Bestatin exerted no significant effect on the proliferation of HRA cells, which expressed a low level of APN/CD13.

To investigate the function of APN/CD13 in OVCA cells, we used bestatin, an APN/CD13 inhibitor, in ES-2 cells, which expressed considerable amounts of APN/CD13. We confirmed that the APN/CD13 activity of ES-2 cells was markedly reduced by bestatin, as demonstrated by reduced absorbance in the enzymatic activity assay (*p *< 0.001) (Fig. 3A). Fig. 3C shows that more than 100 μg/ml of bestatin significantly inhibited the proliferation of ES-2 cells. However, bestatin did not influence the proliferation of HRA cells, which expressed a low level of APN/CD13 (Fig. 3D). We performed the trypan blue dye-exclusion test to evaluate the cytotoxity of the bestatin concentration used. We confirmed that there was no obvious cytotoxic effect of bestatin on HRA cells but that bestatin dose-dependently induced cytotoxicity in ES-2 cells (Fig. 3B).

**Figure 4:**
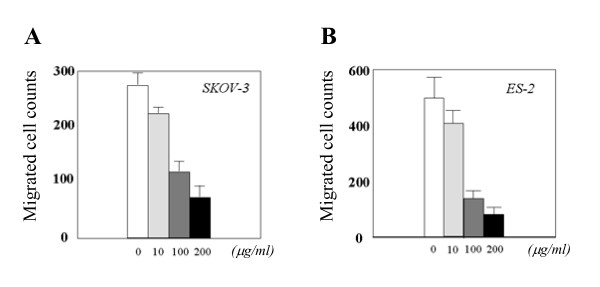
Bestatin inhibited the cell motility of APN/CD13-expressing OVCA cells in the Transwell migration assay in a concentration-dependent manner. A: SKOV-3 cells; *; *p *< 0.01, **; *p *< 0.001, B; ES-2 cells; *; *p *< 0.001, **; *p *< 0.0001.

We also assessed the effect of bestatin on migration in OVCA cells. As shown in Fig. 4, the addition of bestatin significantly inhibited the migration of ES-2 and SKOV-3 cells in the Transwell migration assay in a concentration-dependent manner. In contrast, bestatin did not influence the migration of HRA cells (data not shown).

### Effect of inhibition of APN/CD13 using siRNA on the migration and proliferation of ES-2 cells

**Figure 5:**
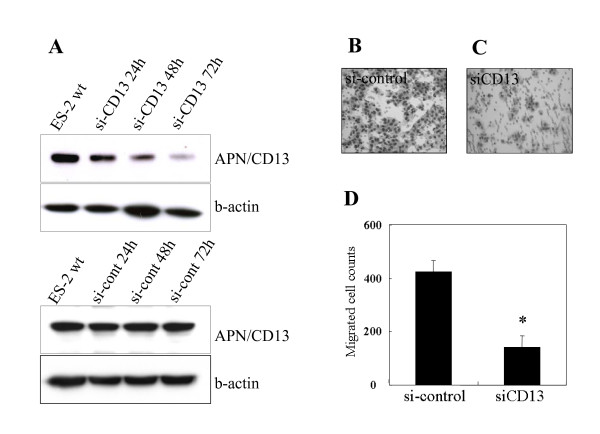
Suppression of APN/CD13 expression by siRNA induced a marked decrease in migratory potential of ES-2 cells. A; Western blot analysis showed a decrease in APN/CD13 expression after siRNA transfection. B, C; Giemsa staining showing the migration of ES-2 cells transfected with non-specific control siRNA (B; si-cont) or siRNA specific for APN/CD13 (C; si-CD13), respectively. D; The level of migration of ES-2 cells transfected with si-CD13 relative to the control was 33%. Data are expressed as the mean ± SD *; *p *< 0.01.

**Figure 6:**
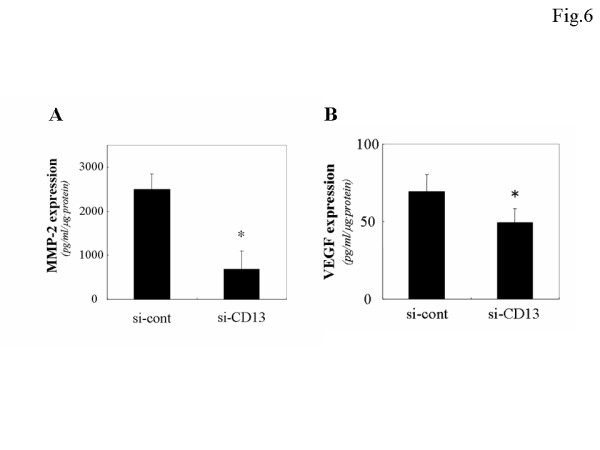
Decrease of MMP-2 and VEGF expression caused by RNA interference of APN/CD13 in ES-2 cells using MMP-2 and VEGF ELISA kit. The MMP-2 and VEGD expression in the conditioned medium of si-CD13-transfected ES-2 cells was significantly lower than that of si-cont-transfected cells. Data are expressed as the mean ± SD, A; *p < 0.001, B; *p < 0.05.

**Figure 7:**
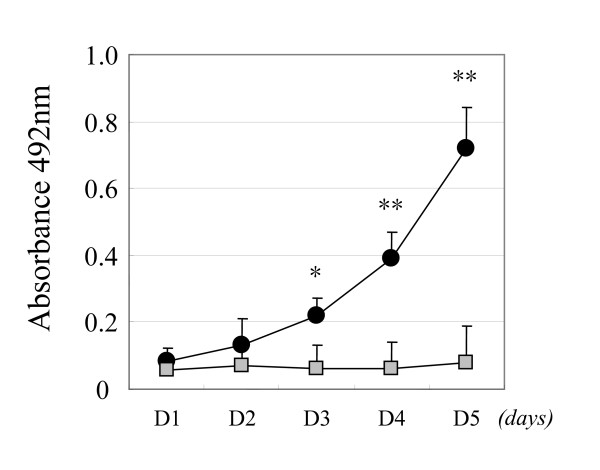
Inhibition of proliferative potential by the transfection of siRNA for APN/CD13 in ES-2 cells in a 4-day modified MTT assay. Analysis of the proliferation rate was performed as described in "MATERIALS AND METHODS". si-CD13-transfected cells showed almost no growth, whereas the si-cont-transfected cells grew similarly to the parental ES-2 cells. *Closed circles*; si-cont-transfected cells, *gray squares*; si-CD13-transfected cells. Data are expressed as the mean ± SD of four independent experiments. *; *p *< 0.01, **; *p *< 0.001.

To further confirm the significance of APN/CD13 expression in the cellular function of OVCA, we examined the effect of APN/CD13 silencing using an siRNA system in ES-2 cells. Two siRNAs (si-CD13 and si-CD13-2) that specifically reduced the APN/CD13 protein level were developed. Since the si-CD13 was more efficient for the reduction of APN/CD13 expression compared with si-CD13-2 (data not shown), we used si-CD13 in the following experiment. Fig. 5A shows Western blot analysis of APN/CD13 expression at 24, 48, and 72 hours post transfection. The APN/CD13 protein level was most efficiently reduced after 72 hours of transfection. At this point, the migratory potential of si-CD13-transfected cells as estimated using the Transwell migration assay was significantly reduced to approximately 30% of that of the si-cont-transfected cells (Fig. 5B). Based on our finding that the migratory potential was downregulated by the silencing of APN/CD13 using siRNA, we next directly examined the effect of APN/CD13 inhibition on the MMP-2 and VEGF expression. The MMP-2 and VEGF expression in the conditioned medium of si-CD13-transfected ES-2 cells was 20.7% and 71.1% lower than that in si-cont-transfected cells, as shown in Figure 6A and 6B, respectively. Furthermore, silencing of APN/CD13 by siRNA resulted in a reduction of the growth rate of ES-2 cells (Fig. 7). The si-cont-transfected cells proliferated at the same rate as the parental ES-2 cells. In contrast, si-CD13-transfected cells could not grow at all.

### The effect of bestatin on the peritoneal progression of ovarian carcinoma cells in a nude mice model

**Figure 8:**
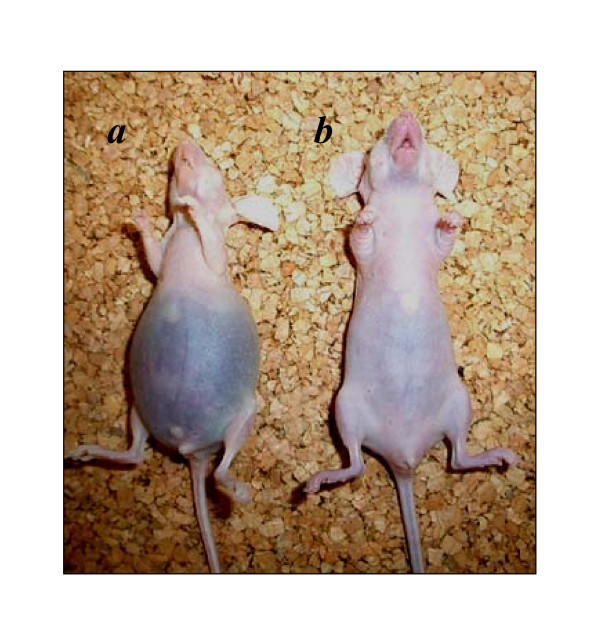
Anti-metastatic effect of bestatin treatment in SKOV-3 cells. A representative example of nude mice 30 days after i.p. inoculation of 1.0 × 10^7 ^of SKOV-3 cells with or without subsequent daily treatment with bestatin, showing a mouse treated with PBS alone (a; *left*) or with bestatin (20 mg/kg, daily)(*b; right*). More apparent symptoms of carcinomatous peritonitis were observed in mice treated with PBS alone than in those treated with bestatin. In this experiment, each group consisted of seven mice.

Finally, we tested whether bestatin influenced the peritoneal dissemination or survival of OVCA using nude mice. We previously confirmed that carcinomatous peritonitis was observed approximately 2 or 4 weeks after the inoculation of HRA or SKOV-3 cells, respectively, in mice. In the current experiments, daily intraperitoneal administration of bestatin after tumor inoculation was performed. The results showed that in the SKOV-3 cell-inoculated group, treatment with bestatin resulted in decreased peritoneal dissemination and prolonged survival of the mice. On the other hand, in the HRA cell-inoculated group, bestatin did not influence peritoneal dissemination or survival (Fig. 8 and Table 3).

**Table 3:** The effect of Bestatin on survival time of nude mice

**Cells**	**Control-group**	**Bestatin-group**
**HRA**	13.8 ± 0.8	15.8 ± 2.5
**SKOV-3**	29.0 ± 3.0	*36.1 ± 6.5 (*days*)

Mean survival days of PBS alone or bestatin treated mice with innoculation of 1.0 × 10^7 ^HRA and SKOV-3 cells, respectively. Data are expressed as the mean ± SD. *; p < 0.05

## Discussion

Cell-surface peptidases play a key role in controlling the growth, differentiation and signal transduction of many cellular systems by modulating the activity of peptide factors and regulating their access to receptors [4]. According to several other reports, APN/CD13 is involved in the enhanced cell motility and invasive ability of tumor cells, and in the neovascularization of endothelial cells [5,21-23]. In the current study, we focused on the expression and function of APN/CD13 in OVCA. We showed that the morphology of APN/CD13-expressing cell lines tended to show a spindle/bipolar fibroblast-like pattern, which was associated with high invasiveness and enhanced MMP-2 expression. In contrast, cells with low expression of APN/CD13 showed an epithelioid pattern. This effect of APN/CD13 on morphology was opposite the effect of DPPIV/CD26 on OVCA cells shown in our previous report [24]. Based on this result, although APN/CD13 and DPPIV/CD26 are both cell surface aminopeptidases, their effects on migratory potential and cell morphology are thought to be opposite. Kehlen *et al*. reported that undifferentiated anaplastic thyroid carcinomas expressed larger amounts of APN/CD13 than differentiated thyroid carcinomas, whereas in contrast, higher levels of DPPIV/CD26 were expressed in follicular and papillary thyroid carcinomas than in undifferentiated anaplastic thyroid carcinomas [23]. Thus, consistent with our results in OVCA, the expression patterns of DPPIV/CD26 and APN/CD13 in thyroid carcinoma were also opposite. Our present data demonstrated that the invasiveness and/or migratory potential are comparatively strong in CD13+/CD26-OVCA cells (*e.g*., ES-2, SKOV-3, and HEY cells); in contrast, these abilities are weak in CD13-/CD26+ OVCA cells (*e.g*., TAOV, NOS2, and NOS4 cells). We think that there may be a crosstalk between APN/CD13 and DPPIV/CD26 expressions in a variety of cells, and that the expression-balance between these two molecules may be critical for the cellular invasive potential, although further studies are needed to confirm this. However, HRA cells are CD13-/CD26-type, and thus their characteristics cannot be explained by the CD13/CD26 balance, and their invasiveness must be based on some other mechanism.

To study the function of APN/CD13 in OVCA, we first used bestatin (Ubenimex), which is a competitive inhibitor of APN/CD13 and an antibiotic with inhibitory activity toward some but not all aminopeptidases [25]. Our current findings demonstrated that the suppression of APN/CD13 activity by bestatin in APN/CD13-expressing cells reduced the migratory and proliferative potential as well as the peritoneal dissemination in a mice model, which led to prolonged survival. On the other hand, in OVCA cells with limited expression of APN/CD13, bestatin had no apparent influence on these cell functions related to tumor progression. These results indicated the direct involvement of APN/CD13 in the tumor-progression of OVCA, which was consistent with the finding of previous reports. Considering these results, APN/CD13 activity may play a crucial role in cell motility and the metastasis of OVCA, since APN/CD13 has been suggested to be involved in the degradation of neuropeptides, cytokines, and immunomodulatory peptides, as well as angiotensins [26,27]. Riemann *et al*. reported that in leukemic cells, APN/CD13 expression may contribute to the malignant phenotype via proteolytically modifying peptides and/or their precursors involved in growth stimulation or retardation [6]. Although bestatin affects the enzymatic activity of APN/CD13, it is not necessarily specific for APN/CD13 and bestatin can directly cause apoptosis in tumor cells besides its ability to inhibit APN/CD13 activity. Therefore, an siRNA method able to down-regulate all functions of APN/CD13 was used in our current *in vitro *examination. The results showed that silencing of APN/CD13 expression by siRNA induced a marked decrease in the migratory and proliferative potential of OVCA cells with a concurrent down-regulation of MMP-2 and VEGF expression. Chan *et al*. provided strong evidence that APN/CD13 activity was not absolutely required for enhanced migration by demonstrating that expression of a catalytically inactive form of APN/CD13 significantly enhanced the migration of lung adenocarcinoma cells [27]. Taken together, our results also support the notion that APN/CD13 may promote cancer cell migration or metastasis by both aminopeptidase-dependent and aminopeptidase-independent mechanisms.

With regard to the effect of APN/CD13 expression on the progression of OVCA, van Hensbergen et al, previously reported that there was no distinct effect of APN/CD13-overexpression on the growth of OVCA cells (IGROV-1 cells) in vitro, but it was associated with decreased invasive ability[28]. In our current study, inhibition of APN/CD13 by siRNA resulted in a reduction of the growth and invasion of OVCA cells, although there was no obvious correlation between the expression of APN/CD13 and doubling time of various OVCA cells. Unfortunately, we can not explain for this discrepancy at present. However, our assay was based on the inhibition of APN/CD13 expression or its activity using siRNA or bestatin, which was somewhat different from their assays using APN/CD13-overexpressing transfectants. In addition, the discrepancy may be due to some peculiarity of IGROV-1 cells, because the work of Hensbergen et al, was derived from the results using this single cell line. Also, the discrepancy might be due to differences of the expression of several unknown co-factors that facilitate the function of APN/CD13. At any rate, to elucidate the mechanistic function of APN/CD13 including these discrepancies, further investigations of APN/CD13 in tumor biology will be essential.

Clinically, several reports have shown that APN/CD13 was correlated with poor prognosis in several malignancies [9,29]. Hashida *et al*. reported that APN/CD13 was involved in cell motility or angiogenesis, and that the expression of this enzyme was associated with poor prognosis for node-positive patients with human colon cancer [9]. According to the recent report from Surowiak et al. [30], there was a slight, although not significant, prognostic difference between patients positive for APN/CD13 expression and those negative for APN/CD13 expression. However, in their study, the majority of the cases were at stage-III (95%). We think that this could have caused some bias. Namely, in many survival analyses including several clinicopathological factors, stage is an independent prognostic factor. If the influence of stage was very strong, the importance of APN/CD13 expression as a prognostic factor might become weak. Thus, a larger number of patients including early stage (I-II) have to be examined in the next analysis of the prognosis of OVCA.

Considering the body of evidence about APN/CD13, including our current results, APN/CD13 may be a target for treatment of OVCA. If effective, bestatin may be one of the candidate drugs with APN/CD13 as a clinical target, and is already being used clinically for the treatment of leukemia. According to a recent report by Ichinose *et al*., survival was significantly better for patients with completely resected stage I squamous-cell lung carcinoma who were treated with bestatin as a postoperative adjuvant therapy than for those who received a placebo [31].

## Conclusion

The data obtained in the current investigation demonstrated the expression APN/CD13 in OVCA associated with tumor cell motility, proliferation and metastasis. Therefore, further insights into the mechanism and function of APN/CD13 in tumor biology may be critical, as APN/CD13 may constitute a potential therapeutic target in a variety of malignancies.

## List of abbreviations

APN: aminopeptidase N, OVCA: ovarian carcinoma.

## Competing interests

The author(s) declare that they have no competing interests.

## Authors' contributions

MT performed all in vitro functional analysis of APN/CD13 using cell lines and interpreted results and drafted the manuscript. HK performed animal experiment and statistical analysis on the result and designed the expression studies. KS carried out the immunoassays. KN performed the immunohistochemical staining, AN and SM conceived of the study, and participated in its design and coordination. FK supervised all work and aided in the drafting of the manuscript. All authors read and approved the final manuscript.

## Pre-publication history

The pre-publication history for this paper can be accessed here:


